# Cardiac troponin and increased mortality risk among individuals with restrictive spirometric pattern on lung function testing

**DOI:** 10.1080/20018525.2024.2436203

**Published:** 2024-12-10

**Authors:** Sara Johansson, Petra Sandin, Lenita Lindgren, Nicholas L Mills, Linnea Hedman, Helena Backman, Ulf Nilsson

**Affiliations:** a Department of Nursing, Umeå University, Umeå, Sweden; b BHF Centre for Cardiovascular Science, University of Edinburgh, Edinburgh, UK; c Usher Institute, University of Edinburgh, Edinburgh, UK; d Department of Public Health and Clinical Medicine, Umeå University, Umeå, Sweden

**Keywords:** Spirometry, cardiac disease, mortality, troponin, natriuretic peptides, epidemiology

## Abstract

**Background:**

Individuals with a restrictive spirometric pattern have a high burden of cardiovascular and metabolic morbidity.

**Objective:**

To assess prevalence of elevated cardiac biomarkers among individuals with a restrictive spirometric pattern compared to those with a normal lung function and to evaluate the association between cardiac biomarkers and mortality.

**Methods:**

In 2002–04, individuals with airway obstruction were identified from population-based cohorts, together with age- and sex-matched non-obstructive referents. The analysis population consisted of the non-obstructive referents stratified according to whether they had a restrictive spirometric pattern or normal lung function in whom cardiac biomarkers were measured. Deaths were recorded until 31 December 2010.

**Results:**

Participants with a restrictive spirometric pattern were older and more likely to be obese with a higher burden of cardiovascular risk factors than those with normal function. Elevated cardiac troponin but not natriuretic peptide levels were more common in those with a restrictive spirometric pattern independent of age, sex, BMI, or risk factors (adjusted OR 1.8, 95% CI 1.29–2.74). At 5 years, death occurred more frequently in participants with restrictive spirometric pattern compared to those with normal function (15.7% [31/197] versus 7.6% [57/751]), with highest mortality rate in those with restriction and elevated cardiac troponin (28.7% [27/94]). Cardiac troponin was independently associated with death among those with a restrictive spirometric pattern (HR 4.91, 95% CI 1.58–15.26) but not in those with normal lung function.

**Conclusion:**

Cardiac troponin was elevated more often in people with a restrictive spirometric pattern in whom it was a strong independent predictor of death.

## Introduction

Restrictive spirometric pattern is identified on a flow-volume chart by performing a dynamic spirometry test. It is defined as a preserved FEV_1_/FVC ratio and a reduced FVC [1]. The prevalence of a restrictive spirometric pattern ranges from 5% to 10% and in certain populations even up to 30–60%, with the higher prevalence occurring mainly in low-income countries [1,2]. Individuals with a restrictive spirometric pattern comprise a heterogenous group with several underlying conditions that may affect the results of a spirometry, for example, thoracic deformity, obesity, neuromuscular disease, cardiac insufficiency, pleural effusion, and interstitial lung disease [3]. It has been reported that individuals with a restrictive spirometric pattern have three to six times higher prevalence of ischemic heart disease, compared to individuals with normal lung function [4,5], but the underlying causes are yet to be explained [2].

Ischemic heart disease can be diagnosed in several ways. For the diagnosis of myocardial damage, a 12-lead ECG and blood analysis of cardiac specific troponin are commonly used. Troponin is released from the myocardium following injury and can be detected within hours [6]. Cardiac troponins have also been shown to be elevated in chronic pulmonary conditions [7,8] and to be associated with an increased mortality risk, even at levels below the thresholds used for the diagnosis of myocardial infarction [9–11]. Whilst N-terminal pro-B-type natriuretic peptide (NT-proBNP) is a cornerstone in the diagnosis of heart failure, it may also serve as a risk predictor for future cardiovascular events and mortality [12].

So far, there are no previous longitudinal population-based studies describing the relationship between cardiac troponin and NT-proBNP levels in individuals with a restrictive spirometric pattern and its association with mortality.

Our aim was to assess the frequency of elevated cardiac troponin and NT-proBNP levels among individuals with a restrictive spirometric pattern compared to those with normal lung function and to evaluate the association between these cardiac biomarkers and mortality in both groups.

## Material and methods

### Study sample

The recruitment of the study population and methods for lung function testing have been described previously [13]. In summary, from 4,200 participants in four population-based cohorts from the Obstructive Lung Disease in Norrbotten (OLIN) studies, 993 individuals were identified as having chronic airway obstruction after a re-examination with reversibility testing in 2002–04. In addition, a reference group of 993 non-obstructive individuals matched by age and sex was recruited from the same cohorts. These 1,986 individuals formed the OLIN COPD study population, which has been invited to annual examinations between 2005 and 2015.

**Figure 1.**
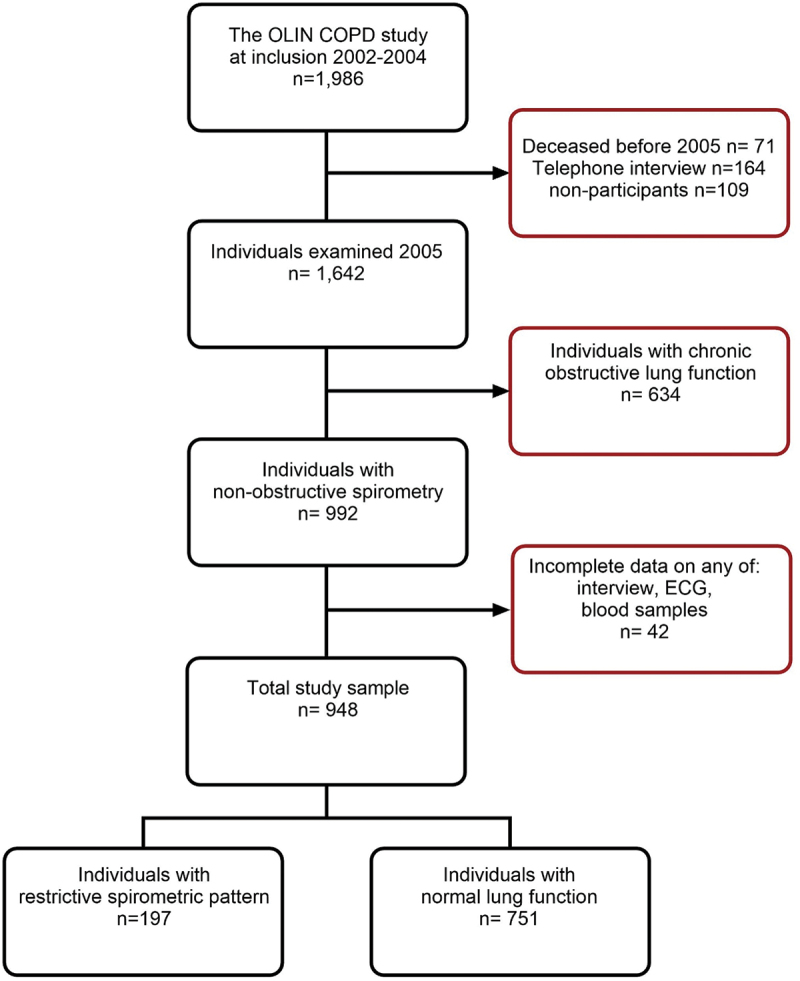
Flow chart describing the selection of study participants and sample.

The present analysis population consists of 948 participants from the reference group without chronic airway obstruction from 2005, in whom blood samples and an electrocardiogram were collected along with spirometry and structured interviews. The group was further stratified into individuals with a restrictive spirometric pattern (FEV_1_/VC > 0.70 and VC < 80%) (*n* = 197) and individuals with a normal lung function (FEV_1_/VC > 0.70 and VC > 80%) (*n* = 751) (Figure 1). Local reference values based on healthy non-smokers were used [14]. Mortality data were collected from the Swedish Tax Agency from the date of examination in 2005 extending until 31 December 2010. Cardiovascular mortality was defined as any ICD-10 code I00-I99 registered as the underlying cause of death on the death certificate. The study was approved by the Regional Ethical Review Board at Umeå University, Sweden, project approval numbers: Um dnr 04–045 M and 2015/446–31Ö. Written informed consent was obtained from all participants.

### Measurements

Blood samples were stored at −20**°**C, thawed and analysed for cardiac troponin measurement using the ARCHITECT *STAT* high-sensitivity cardiac troponin I assay (Abbott Laboratories, Abbott Park, IL, USA) in the BHF Cardiovascular Biomarker Laboratory at the University of Edinburgh, Edinburgh. The assay has a lower limit of detection (LoD) of 1.2 ng/L, an inter-assay coefficient of variation of less than 10% at 4.7 ng/L, and sex-specific 99th centile upper reference limits of 34 ng/L in men and 16 ng/L in women [15]. Cardiac troponin concentrations above the risk stratification threshold of 5 ng/L were defined as elevated [11]. NT-proBNP was measured using an electrochemiluminescence method with the R-PLEX® human NT-proBNP antibody set (Meso Scale Diagnostics, Rockville, MD, USA) at the Respiratory research lab, Umeå University, Umeå, Sweden, according to instructions from the manufacturer. The lower limit of detection was 0.3 pg/mL, and a risk stratification threshold was set to 125 pg/mL [16]. Observed values below LoD for both biomarkers were included in the analyses. Estimated glomerulus filtration rate (eGFR) was calculated using the CKD-EPI 2021 formula [17]. A cutoff for renal failure was set to <60 mmol/min/1.73 m^2^ [18].

Body mass index (BMI) was calculated based on height and weight as kg/m^2^ and categorized as underweight <18.5, normal weight 18.5–24.9, overweight 25–29.9, and obesity ≥30. Pack-years were calculated by multiplying the number of packs of cigarettes smoked per day by the number of years the person has smoked.

### Electrocardiogram

Ischemia on electrocardiogram (ECG) was defined by the presence of either of major Q/QS wave, major isolated ST-T abnormality, minor Q-wave in combination with major ST-T and minor isolated Q wave, using the Minnesota code criteria [19].

### Statistics

Statistical analyses were performed using the SPSS version 28 (IBM, Armonk, NY, USA). Categorical variables were compared using chi-square test, independent sample t-test to compare means and Mann–Whitney U-test to compare medians. Known risk factors for cardiac troponin I above the risk stratification threshold of 5 ng/L were analyzed using unadjusted and adjusted logistic regression and presented as odds ratios (OR) with 95% confidence intervals (CI). Survival in each of the groups was examined by plotting Kaplan–Meier curves. Both cardiac troponin and NT-proBNP were evaluated as risk factors for all-cause mortality and expressed as hazard ratios (HR) with 95% CI in unadjusted and adjusted Cox regression analyses. Continuous variables with skewed distributions were analysed either by applying a risk threshold value or transformed using natural logarithms. P-values of <0.05 were considered significant.

## Results

### Clinical characteristics

Individuals with a restrictive spirometric pattern were older and had greater metabolic burden, with a higher prevalence of diabetes mellitus and a higher mean body mass index (BMI), with 33.0% of the group being obese, compared to 20.9% among individuals with normal lung function. Among individuals with a restrictive spirometric pattern, the prevalence of reported cardiovascular disease and ischemic changes in the ECG was higher compared to the normal lung function group (Table 1).

**Table 1.** Clinical characteristics of the participants with restrictive spirometric pattern (RSP) and normal lung function (NLF).

	RSP n = 197	NLF n = 751	P-value
Male sex n(%)	111 (56.3)	393 (52.3)	0.366
Age, years mean (SD)	**68.9 (10.1)**	**64.5 (11.3)**	**<0.001**
BMI, kg/m^2^ mean (SD)	**28.8 (4.8)**	**27.0 (4.0)**	**0.038**
BMI normal n(%)	37 (18.8)	248 (33.0)	–
BMI overweight n(%)	94 (47.7)	344 (45.8)	–
BMI obese n(%)	65 (33.0)	157 (20.9)	–
BMI underweight n(%)	1 (0.5)	2 (0.3)	–
Diabetes mellitus n(%)	**41 (20.6)**	**64 (8.5)**	**0.001**
Current smoker n(%)	21 (10.7)	97 (12.9)	0.393
Pack-years mean (SD)	8.5 (13.1)	7.0 (10.8)	0.138
Heart failure n(%)	**9 (4.5)**	**7 (0.9)**	**0.001**
Angina pectoris n(%)	**40 (20.1)**	**80 (10.6)**	**0.001**
Myocardial infarction n(%)	**15 (7.5)**	**19 (2.5)**	**0.001**
CABG and/or PCI n(%)	**19 (9.5)**	**34 (4.5)**	**0.006**
Ischemic heart disease^a^ n(%)	**50 (25.1)**	**94 (12.5)**	**<0.001**
Ischemic ECG^b^ n(%)	**38 (19.1)**	**101 (13.4)**	**0.042**
Cardiac troponin ng/L median (IQR)	**4.6 (4.6)**	**3.2 (2.9)**	**<0.001**
Cardiac troponin ≥ 5, ng/L n(%)	**95 (47.7)**	**188 (24.9)**	**0.001**
NT-proBNP, pg/mL median (IQR)	1.2 (14.1)	1.6 (7.1)	0.263
NT-proBNP ≥125, pg/mL n(%)	**12 (6.1)**	**18 (2.4)**	**0.008**
eGFR, mmol/min/1.73 m^2^ median (SD)	**92.7 (20.3)**	**95.6 (17.8)**	**<0.001**
eGFR, <60 mmol/min/1.73 m^2^ n(%)	**14 (7.1)**	**24 (3.2)**	**0.013**
FVC, L mean (SD)	**2.7 (0.7)**	**3.7 (1.0)**	**<0.001**

Significant values (p < 0.05) in bold.

^a^Including angina pectoris, myocardial infarction, coronary artery bypass grafting (CABG) and/or percutaneous coronary intervention (PCI).

^b^Including Major Q/QS wave, major isolated ST-T abnormality, Minor Q wave plus major ST-T and minor isolated Q wave based on Minnesota coding.

BMI: body mass index; eGFR: Estimated glomerular filtration rate; FVC: Forced vital capacity.

### Factors associated with elevated cardiac troponin

The median (inter-quartile range) cardiac troponin concentration in individuals with a restrictive spirometric pattern was higher compared to in individuals with a normal lung function (4.6 [3.0 to 7.6] versus 3.2 [2.1 to 5.0] ng/L, *p* < 0.001) and the number of individuals with a cardiac troponin concentration  ≥ 5 ng/L was two-fold greater among those with a restrictive spirometric pattern compared to normal lung function (47.7% *versus* 24.9%, *p* < 0.001) (Table 1). In a logistic regression model adjusting for age, sex, BMI, and known cardiovascular risk factors, a restrictive spirometric pattern was independently associated with elevated cardiac troponin (OR 1.88, 95% CI 1.29–2.74) (supplemental material, table A1). In contrast, there was no difference in median concentrations of NT-proBNP between the restrictive spirometric pattern and normal lung function groups (2.0 versus 1.6 pg/mL, *p* = 0.26), although the prevalence of elevated NT-proBNP concentration >125 pg/mL was higher among those with a restrictive spirometric pattern compared with normal lung function (6.1% versus 2.4%, *p* = 0.008). Among individuals with a restrictive spirometric pattern, the group with elevated NT-proBNP were older compared to the group without elevated levels (80.1 *versus* 68.1 years, *p* = 0.01), while no significant difference in age was observed in individuals with normal lung function stratified by NT-ProBNP (75.9 *versus* 64.3 years, *p* = 0.084) or between men or women in either group. In a multivariable regression model adjusting for age, sex, and cardiovascular risk factors, restrictive spirometric pattern was not associated with elevated NT-proBNP (OR 1.76, 95% CI 0.79–3.92) (supplemental material, table A2).

In an unadjusted analysis, age, history of ischemic heart disease, NT-proBNP and eGFR <60 mmol/min/1.73 m^2^ were all associated with an elevated cardiac troponin concentration (≥5 ng/L) in both the restrictive spirometric pattern and normal lung function groups (Table 2). In those with a restrictive spirometric pattern, a lower FVC was associated with elevated troponin. Among individuals with a normal lung function, male sex, diabetes mellitus, obesity, and ischemic ECG abnormalities were associated with an elevated cardiac troponin, whereas smoking status and pack-years were inversely related to elevated cardiac troponin (Table 2). In an adjusted model, male sex, NT-proBNP and lower FVC remained significantly associated with elevated cardiac troponin in individuals with a restrictive spirometric pattern. The same pattern was seen among individuals with a normal lung function, where also an association with ischemic ECG abnormalities, eGFR <60 mmol/min/1.73 m^2^ and pack-years was found (Table 2, model 1). When age and BMI was added to the model, male sex, age and NT-proBNP remained associated with cardiac troponin, while the association with FVC was lost in both groups. Among the normal lung function group, ischemic ECG abnormalities, obesity and pack-years remained associated with elevated cardiac troponin (Table 2, model 2).

**Table 2.** Unadjusted and adjusted regression models of features associated with elevated cardiac troponin concentration in participants with restrictive spirometric pattern (RSP) and normal lung function (NLF).

	Unadjusted				Adjusted							
					Model 1				Model 2			
	RSP		NLF		RSP		NLF		RSP		NLF	
	OR	95% CI	OR	95% CI	OR	95% CI	OR	95% CI	OR	95% CI	OR	95% CI
Male sex	1.29	0.73–2.26	**2.40**	**1.69–3.40**	**7.86**	**2.72–22.70**	**5.32**	**2.98–9.48**	**4.49**	**1.28–15.79**	**2.47**	**1.28–4.76**
Age, years	**1.12**	**1.07–1.16**	**1.09**	**1.07–1.11**					**1.08**	**1.02–1.14**	**1.09**	**1.06–1.12**
BMI Normal	Ref		Ref						Ref		Ref	
BMI Overweight	1.95	0.90–4.24	1.23	0.83–1.81					**4.13**	**1.50–11.35**	1.18	0.74–1.88
BMI Obese	1.24	0.54–2.84	**1.62**	**1.03–2.56**					2.63	0.87–7.94	**2.50**	**1.42–4.39**
BMI Underweight	n/a	n/a	3.68	0.23–59.81					n/a	n/a	2.39	0.02–245.53
Current smoker	0.80	0.32–2.00	**0.38**	**0.20–0.72**	1.47	0.44–4.87	0.65	0.30–1.38	2.49	0.68–9.15	0.98	0.45–2.14
Pack-years	1.00	0.98–1.02	**0.98**	**0.96–0.99**	0.99	0.96–1.02	**0.98**	**0.96–1.00**	0.99	0.96–1.02	**0.97**	**0.95–1.00**
FVC, L	**0.47**	**0.30–0.72**	0.94	0.79–1.12	**0.19**	**0.09–0.42**	**0.68**	**0.51–0.92**	0.40	0.14–1.13	1.40	0.97–2.04
Diabetes mellitus	1.53	0.77–3.06	**2.64**	**1.56–4.47**	1.16	0.49–2.74	1.71	0.92–3.16	1.35	0.55–3.28	1.67	0.88–3.17
Ischemic heart disease^a^	**2.44**	**1.26–4.73**	**2.55**	**1.63–3.99**	1.11	0.49–2.51	1.27	0.74–2.18	0.93	0.39–2.19	1.09	0.63–1.91
Ischemic ECG^b^	2.05	0.99–4.29	**3.64**	**2.36–5.61**	1.64	0.68–3.93	**3.75**	**2.26–6.21**	1.72	0.70–4.21	**3.36**	**1.98–5.70**
Ln NT-proBNP	**1.33**	**1.16–1.52**	**1.41**	**1.29–1.54**	**1.23**	**1.05–1.43**	**1.30**	**1.18–1.44**	**1.25**	**1.05–1.48**	**1.22**	**1.10–1.36**
eGFR <60 mmol/min/1.73 m^2^	**4.42**	**1.19–16.36**	**3.73**	**1.64–8.47**	2.80	0.64–12.30	**2.64**	**1.03–6.75**	2.92	0.66–12.95	1.52	0.55–4.22

^a^Including angina pectoris, myocardial infarction, coronary artery bypass grafting (CABG) and/or percutaneous coronary intervention (PCI).

^b^Including Major Q/QS wave, major isolated ST-T abnormality, Minor Q wave plus major ST-T and minor isolated Q-wave based on Minnesota coding.

BMI: Body mass index; eGFR: Estimated glomerular filtration rate; FVC: Forced vital capacity; Ln: natural logarithm.

Significant values (*p* < 0.05) in bold.

### Cardiovascular biomarkers and mortality

**Figure 2.**
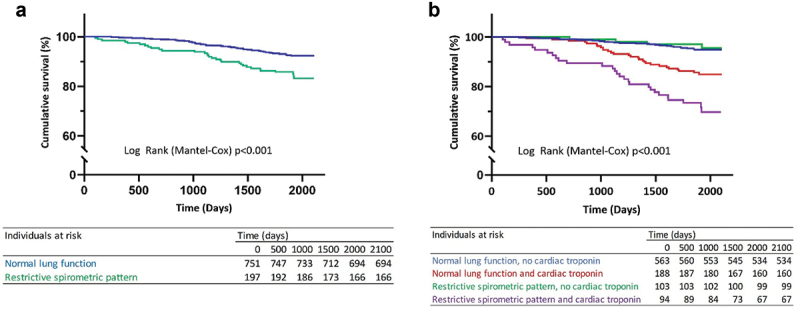
(a) Kaplan–Meier survival curves stratified for restrictive spirometric pattern and normal lung function. (b). Kaplan–Meier survival curves stratified for restrictive spirometric pattern and normal lung function with and without elevated cardiac troponin.

The 5-year cumulative mortality was 15.7% (*n* = 31) among individuals with a restrictive spirometric pattern and 7.6% (*n* = 57) in the normal lung function group (*p* < 0.001) (Figure 2(a)), corresponding to a mortality rate of 31.3 and 14.5 deaths per 1,000 person-years in the restrictive spirometric pattern and normal lung function groups, respectively. In analyses stratified for lung function and elevated cardiac troponin levels, there was a marked increase in the cumulative mortality for individuals with elevated troponin compared to those with normal levels in both the restrictive spirometric pattern (28.7% vs 3.9%, *p* < 0.001) and the normal lung function groups (14.9% vs. 5.2%, *p* < 0.001) (Figure 2(b)). There was no difference in the prevalence of cardiovascular causes of death for individuals with elevated troponin when comparing restrictive spirometric pattern to normal lung function (*n* = 19, 70.4% and *n* = 19, 67.9%, (*p* = 0.84) respectively). However, within the restrictive spirometric group, there were a significant correlation between elevated troponin and cardiovascular mortality (*n* = 19, 100.0%, *p* = 0.02). This correlation was not observed among individuals with normal lung function (*n* = 19, 54.3% *p* = 0.32).

In an adjusted Cox regression model, individuals with restrictive spirometric pattern and elevated troponin had an increased mortality risk compared to those with normal lung function (HR 1.96, 95% CI 1.08–3.52). There was no increased mortality risk observed for individuals with normal lung function with elevated troponin or individuals with restrictive spirometric pattern without elevated troponin (Table 3).

**Table 3.** Unadjusted and adjusted Cox regression analysis of factors associated with mortality in the whole study population.

	Unadjusted		Adjusted	
	HR	95% CI	HR	95% CI
Male sex	**1.84**	**1.18–2.87**	**1.67**	**1.03–2.71**
Age	**1.10**	**1.08–1.13**	**1.07**	**1.04–1.10**
Current smoker	1.13	0.62–2.08		
pack-years	**1.03**	**1.01–1.04**	**1.03**	**1.02–1.05**
BMI normal	ref		Ref	
BMI overweight	**1.71**	**1.01–2.90**	1.51	0.88–2.61
BMI obese	1.30	0.69–2.45	1.35	0.69–2.62
BMI underweight	6.03	0.81–45.03	2.94	0.36–32.77
Ischemic heart disease^a^	**3.90**	**2.54–5.99**	1.61	0.99–2.58
Diabetes mellitus	**2.06**	**1.21–3.49**	0.94	0.52–1.69
Ln NT-proBNP	**1.46**	**1.32–1.61**	**1.24**	**1.11–1.38**
Ischemic ECG^b^	**2.30**	**1.44–3.67**	**1.36**	**0.83–2.24**
eGFR <60 mmol/min/1.73 m^2^	**3.19**	**1.65–6.16**	1.13	0.56–2.29
Normal lung function, no cardiac troponin	ref		ref	
Normal lung function and cardiac troponin	**3.03**	**1.80–5.10**	1.09	0.60–1.98
Restrictive spirometric pattern, no cardiac troponin	0.75	0.26–2.12	0.53	0.18–1.52
Restrictive spirometric pattern and cardiac troponin	**6.50**	**3.85–10.98**	**1.96**	**1.08–3.52**

^a^Including angina pectoris, myocardial infarction, coronary artery bypass grafting (CABG) and/or percutaneous coronary intervention (PCI). ^b^Including Major Q/QS wave, major isolated ST-T abnormality, Minor Q wave plus major ST-T and minor isolated Q-wave based on Minnesota coding.

BMI: body mass index; eGFR: Estimated glomerular filtration rate; Ln: natural logarithm.

Significant values (*p* < 0.05) in bold.

In an unadjusted Cox regression analysis stratified for restrictive spirometric pattern and normal lung function, elevated cardiac troponin was associated with higher mortality in both individuals with a restrictive spirometric pattern (HR 8.68, 95% CI 3.04–24.8) and normal lung function (HR 3.05, 95% CI 1.81–5.12). In a model adjusting for possible confounders, the association remained significant but was attenuated in the restrictive spirometric pattern group (HR 4.91, 95% CI 1.58–15.26) and was lost in normal lung function. However, ischemic ECG and NT-proBNP remained associated with increased mortality risk among individuals with a normal lung function (Table 4).

**Table 4.** Unadjusted and adjusted analyses of risk factors for all-cause mortality stratified for restrictive spirometric pattern (RSP) and normal lung function (NLF).

	Unadjusted				Adjusted			
	RSP		NLF		RSP		NLF	
	HR	95% CI	HR	95% CI	HR	95% CI	HR	95% CI
Male sex	**1.25**	**0.61–2.58**	**2.21**	**1.26–3.90**	**4.69**	**1.16–18.92**	2.13	0.88–5.17
Age	**1.08**	**1.04–1.13**	**1.01**	**1.08–1.14**	1.02	0.95–1.08	**1.09**	**1.05–1.14**
Current smoker	**2.80**	**1.20–6.50**	0.64	0.26–1.60	**9.23**	**3.03–28.09**	1.84	0.69–4.93
BMI normal	ref	–	–	–	–	–	–	–
BMI overweight	0.99	0.42–2.38	1.89	0.97–3.85	–	–	–	–
BMI obese	0.46	0.16–1.37	1.76	0.80–3.85	–	–	–	–
BMI underweight	n/a	n/a	**14.19**	**1.84–109.36**	–	–	–	–
Ischemic heart disease^a^	**2.40**	**1.17–4.90**	**4.47**	**2.61–7.66**	1.41	0.67–2.99	**2.37**	**1.34–4.18**
Diabetes mellitus	1.14	0.49–2.65	**2.48**	**1.25–4.91**	0.75	0.29–1.94	1.28	0.63–2.63
Cardiac troponin ≥ 5 ng/L	**8.68**	**3.04–24.82**	**3.05**	**1.81–5.12**	**4.91**	**1.58–15.26**	0.74	0.39–1.38
Ln NT-proBNP	**1.31**	**1.13–1.53**	**1.51**	**1.33–1.72**	1.15	0.98–1.36	**1.23**	**1.06–1.42**
Ischemic ECG^b^	1.05	0.43–2.56	**3.12**	**1.79–5.46**	0.47	0.18–1.29	**1.97**	**1.08–3.59**
eGFR <60 mmol/min/1.73 m^2^	2.59	0.99–6.74	**3.00**	**1.20–7.51**	1.67	0.57–4.86	0.96	0.36–2.55
FVC, L	**0.48**	**0.28–0.81**	0.77	0.58–1.02	**0.27**	**0.08–0.88**	1.17	0.68–2.01

^a^Including angina pectoris, myocardial infarction, coronary artery bypass grafting (CABG) and/or percutaneous coronary intervention (PCI). ^b^Including Major Q/QS wave, major isolated ST-T abnormality, Minor Q wave plus major ST-T and minor isolated Q-wave based on Minnesota coding.

BMI: body mass index; eGFR: Estimated glomerular filtration rate; FVC: Forced vital capacity; Ln: natural logarithm.

Significant values (*p* < 0.05) in bold.

## Discussion

This study is, to the best of our knowledge, one of the first prospective epidemiological studies of cardiac biomarkers and prognosis in individuals with a restrictive spirometric pattern. Our findings demonstrate that individuals with a restrictive spirometric pattern had higher cardiac troponin concentrations and were twice as likely to have values above established risk stratification thresholds than individuals with a normal lung function. Higher levels of NT-proBNP as well as age were associated with elevated cardiac troponin among both those with and without a restrictive spirometric pattern. However, elevated cardiac troponin was independently associated with mortality only among individuals with a restrictive spirometric pattern.

We found that individuals with a restrictive spirometric pattern on a dynamic spirometry comprised a heterogeneous group with several different underlying non-pulmonary medical conditions. Compared to those with normal lung function, we observed a higher prevalence of cardiovascular disease, raised biomarker levels and ischemic ECG changes in the restrictive spirometric pattern group, in which also a higher mean BMI and a higher prevalence of diabetes mellitus were found, in line with previous studies [5,20]. The restrictive spirometric pattern group has been found to have a partial overlap with a true restriction, as defined by a decreased total lung capacity [2,21]. In a population-based cohort from the Swedish SCAPIS study, it was found that a restrictive spirometric pattern works as a low validity proxy for true pulmonary restriction and is a useful tool for ruling out true pulmonary restriction [22]. On a population level, individuals with a restrictive spirometric pattern carry a high-risk profile for metabolic and cardiovascular diseases and, in line with our results, several previous studies have described their overall risk of comorbidities and mortality [2,4,5,23]. The findings add additional knowledge by describing the importance of cardiovascular biomarkers at subclinical levels as risk markers for death among individuals with a restrictive spirometric pattern.

The clinical utility of troponin in cardiovascular healthcare has changed over the years. With the introduction of high-sensitivity assays, troponins have moved from being a diagnostic marker to also function as a tool for triage. The 99th percentile upper reference limit for troponin I recommended for the diagnosis of myocardial infarction is 34 ng/L for men and 16 ng/L for women [15]. Lower concentrations have been recommended for risk stratification in the general population [24]. In one meta-analysis on more than 22,000 patients, individuals with a cardiac troponin I concentration of less than 5 ng/L were at low risk for cardiac events in both the short and long term [25], whereas another population-based study identified 5.9 ng/L as a useful threshold for an increased cardiovascular risk [10], and others have proposed the use of sex-specific risk stratification threshold [24].

Our data showed that individuals with a restrictive spirometric pattern had a much higher prevalence of elevated troponin I compared to individuals with a normal lung function and that the association between restrictive spirometric pattern and elevated troponin remained even after adjusting for other known risk factors. FVC was inversely related to elevated troponin among both restrictive spirometric pattern and normal lung function individuals, but when age was added to the regression models, the association between FVC and elevated cardiac troponin was lost in both groups. This might be explained by the normal decline in FVC with increasing age [26], as was also found in the present study, in combination with an increase in cardiovascular morbidity with increasing age [27]. The underlying causes of cardiovascular disease and elevated cardiac troponin in individuals with a restrictive spirometric pattern have not been thoroughly established. However, both subclinical and clinical cardiovascular disease could be caused by several factors related to restrictive pulmonary conditions and associated comorbidities. Previous studies have reported elevated levels of systemic inflammatory markers in individuals with restrictive spirometric pattern. Systemic inflammation is a known contributing factor to atherosclerosis and atherothrombosis and may originate from various lung diseases as well as obesity and diabetes [28]. Increased platelet aggregability due to hypoxia has been described among COPD patients, and it is possible that transient hypoxia in individuals with a restrictive spirometric pattern could play a role in activation of thrombotic formation on a subclinical and clinical level [28]. In this study, the high prevalence of obesity and diabetes mellitus along with other factors related to a restrictive spirometric pattern may partly explain the high prevalence of ischemic heart disease.

Regarding NTproBNP, the threshold of 125 pg/mL that was used in this study has been defined as a reference level for ruling out heart failure among individuals in non-acute situations by the European Society of Cardiology [29,30]. A cross-sectional population-based study described that higher age and female sex might contribute to an increased prevalence of NTproBNP above 125 pg/mL [16]. In our study, there was a low prevalence of elevated NTproBNP, with no difference related to sex. However, individuals with elevated NTproBNP levels in the restrictive spirometric pattern group were older and had an increased burden of cardiovascular disease which might explain the higher prevalence.

We observed an inverse association between smoking and elevated cardiac troponin I in the normal lung function group. Smoking is a potent risk factor for cardiovascular disease and could be expected to be related to higher troponin levels. However, an inverse relationship between smoking status and cardiac troponin has been observed in other general population studies [31,32]. One explanation here could be that smokers without symptoms continue to smoke, while smokers with cardiovascular symptoms or disease quit smoking to a larger extent due to secondary prevention interventions [33]. Another possible reason may be the phenomenon of smoking as a protective factor for cardiac health, called the smoker’s paradox. It is considered controversial but has been described in other studies, including patients with acute myocardial infarction, heart failure and stroke [31,34]. Whilst the possible mechanisms behind this finding are not clear, smoking cessation is still a very important public health measure.

In this study, the mortality rate among individuals with a restrictive spirometric pattern was more than twice as high compared to among those with a normal lung function and in line with previous findings [35]. A majority of the deceased in both groups had elevated cardiac troponin at baseline and, in an adjusted model, troponin was independently associated with a five times higher mortality risk in individuals with a restrictive spirometric pattern. This finding of cardiac troponin as an independent predictor of all-cause mortality is in line with other reports from selected as well as in general populations [24,36]. However, the association between all-cause mortality and elevated cardiac troponin in restrictive spirometric pattern has not previously been described. Among individuals with elevated cardiac troponin, cardiovascular causes of death were common in both spirometric groups. However, the correlation between elevated troponin and cardiovascular causes of death was observed only in those among individuals with a restrictive spirometric pattern. This may be related to the high prevalence of ischemic heart disease in that group, although other cardiovascular causes could also be involved. For example, heart failure was more prevalent in the restrictive group, but the available data did not specify whether it was connected to the left or right side of the heart. Right-sided heart failure can be associated with pulmonary disease. Due to the relatively low number of deceased individuals in each spirometric group, a more detailed analysis on specific causes of cardiovascular death could not be performed.

A population-based meta-analysis reported that the addition of troponin I > 6 ng/L to other known risk factors improves the risk prediction for mortality and cardiovascular events among individuals without known cardiovascular disease [24]. In a study on patients with chest pain, troponin T levels above 5 ng/L showed a graded increase in the association between stratified troponin levels and the 3-year risk of all-cause mortality, with a hazard ratio of 2.0 for troponin T levels between 5 and 9 ng/L [36]. Several studies have also shown the predictive value of troponin in pulmonary disease. Elevated troponin levels in patients with acute exacerbation of COPD were related to an increased mortality risk [37]. A similarly enhanced mortality risk has also been seen in individuals with stable COPD [9,10], as well as in individuals early in the disease process of COPD and without any known cardiovascular disease [9]. Troponin seems to be a valid risk marker for mortality in both general populations and among individuals with stable as well as acute exacerbations of COPD. Our findings show that troponin is a risk marker for mortality also among individuals with restrictive spirometric pattern.

The strengths of this study are the use of well-validated methods for data collection on a non-obstructive control group identified from population-based cohorts. Other strengths include the use of a structured interview, a well-validated high-sensitivity cardiac troponin I assay as well as spirometry performed in accordance with international standards and by experienced staff. There are some limitations that merit further discussion. The use of a non-obstructive control group that initially were matched in age and sex to a population-based cohort with chronic airway obstruction could potentially affect the distribution of age and sex in the sample in this study. However, the prevalence of restrictive spirometric pattern was in line with other population-based studies, and individuals in the control group were recruited from four large population-based cohorts which gave the possibility to a detailed clinical characterization of each individual. The relatively small group of individuals with a restrictive spirometric pattern imposes certain statistical limitations for subgroup analyses and some of the findings should therefore be interpreted with caution. The small number of deceased individuals, along with Swedish register data on cause of death may have some uncertainties due to the standard procedure in Sweden to base death certificates on clinical diagnoses, not postmortem autopsy, also limited the ability to conduct more in-depth analyses of specific causes of death. The blood samples were initially stored at −20°C, but we do not anticipate that storage has affected the troponin concentrations, as detectable troponin levels have been found in >99% of a study population with samples stored for 20 years [38]. Several conditions besides ischemic heart disease can cause elevated troponin, for example, atrial fibrillation, stroke and valvular heart disease as well as non-cardiac factors such as age, renal function, and sex [29]. It cannot be ruled out that factors like these may have influenced the results. However, by adjusting the analyses for possible confounders and using troponin I, which is more cardio-specific than troponin T [29], we have mitigated the Impact of these confounding factors. Further research is warranted to explore the pathophysiological mechanisms underlying the high prevalence of elevated levels of troponin, cardiovascular disease and cardiovascular mortality in restrictive spirometric pattern.

NT-proBNP levels have been described to show some sensitivity to freezing and thawing of the blood samples [39,40]. The blood samples used for analysis of NT-proBNP here were all kept continuously frozen since collected, and all samples were analyzed on the same occasion. We cannot rule out that the 18-year of storage in combination with thawing might have affected the overall levels of NT-proBNP; however, the potential effect of handling and storage on NT-proBNP concentrations would be similar for all samples.

The epidemiological design of the study, along with these limitations, restricts the direct clinical applicability of the results. However, the results highlight the need for cardiovascular risk assessment in the meeting with patients with restrictive spirometric pattern. For troponin to be effectively integrated into the risk assessment in a clinical setting, all available clinical information needs to be taken into consideration.

## Conclusions

In this study, individuals with a restrictive spirometric pattern had higher concentrations of cardiac troponin I and NT-proBNP compared to individuals with a normal lung function. The restrictive spirometric pattern-group had a higher mortality rate, and individuals with a restrictive spirometric pattern in combination with elevated cardiac troponin had the worst prognosis. This association was not seen for elevated proBNP. Taken together, these findings suggest that individuals with a restrictive spirometric pattern should be considered for further cardiovascular risk evaluation.

## Supplementary Material

Supplemental Material
