# Supplementary material for: Cardiac troponin and increased mortality risk among individuals with restrictive spirometric pattern on lung function testing
Source: Eur Clin Respir J. 2024 Dec 10;12(1):2436203. doi: 10.1080/20018525.2024.2436203 (PMC11633418; doi:10.1080/20018525.2024.2436203)
Supplement: Supplemental Material [file ZECR_A_2436203_SM2573.docx]

Appendix. Supplementary material

**Supplemental Table A1. Adjusted logistic regression analysis of features associated with elevated cardiac troponin in the total study population.**

|  |  |  |
| --- | --- | --- |
|  | **OR** | **95% CI** |
| Male sex | **2.78** | **1.98-3.90** |
| Age | **1.10** | **1.08-1.12** |
| BMI Normal | **Ref** |  |
| BMI Overweight | 1.25 | 0.84-1.85 |
| BMI Obese | **1.64** | **1.03-2.61** |
| BMI Underweight | 3.92 | 0.17-88.99 |
| Diabetes mellitus | 1.48 | 0.91-2.39 |
| Current smoker | 0.91 | 0.51-1.61 |
| Ischemic heart disease^1^ | 1.11 | 0.72-1.72 |
| Ischemic ECG^2^ | **2.49** | **1.62-3.85** |
| Restrictive spirometric pattern | **1.88** | **1.29-2.74** |
| eGFR <60 ml/min/1.73m | 1.97 | 0.91-4.26 |
| ^1^Including angina pectoris, myocardial infarction, coronary artery bypass grafting (CABG) and/or percutaneous coronary  intervention (PCI). ^2^Including Major Q/QS wave, major isolated ST-T abnormality, Minor Q wave plus major ST-T and minor isolated Q-wave based on Minnesota coding  BMI: Body mass index; eGFR: Estimated glomerular filtration rate; FVC: Forced vital capacity; Ln: natural logarithm  Significant values (p<0.05) in bold. | | |

**Supplemental Table A2. Adjusted logistic regression analysis of features associated with elevated NT-proBNP in the total study population.**

|  |  |  |
| --- | --- | --- |
|  | **OR** | **95% CI** |
| Male sex | 1.30 | 0.59-2.87 |
| Age | **1.14** | **1.08-1.20** |
| BMI Normal | **ref** |  |
| BMI Overweight | 1.22 | 0.50-3.03 |
| BMI Obese | 0.71 | 0.21-2.40 |
| BMI Underweight | **n/a** | **n/a** |
| Diabetes mellitus | **2.62** | **1.07-6.44** |
| Current smoker | 1.68 | 0.34-8.19 |
| Ischemic heart disease^1^ | 1.65 | 0.71-3.81 |
| Ischemic ECG^2^ | 1.00 | 0.39-2.57 |
| Restrictive spirometric pattern | 1.76 | 0.79-3.92 |
| eGFR <60 ml/min/1.73m | 1.92 | 0.63-5.87 |
| ^1^Including angina pectoris, myocardial infarction, coronary artery bypass grafting (CABG) and/or percutaneous coronary  intervention (PCI). ^2^Including Major Q/QS wave, major isolated ST-T abnormality, Minor Q wave plus major ST-T and minor isolated Q-wave based on Minnesota coding  BMI: Body mass index; eGFR: Estimated glomerular filtration rate; FVC: Forced vital capacity; Ln: natural logarithm  Significant values (p<0.05) in bold. | | |
